# Fasting hepatic de novo lipogenesis is not reliably assessed using circulating fatty acid markers

**DOI:** 10.1093/ajcn/nqy304

**Published:** 2019-02-05

**Authors:** Fredrik Rosqvist, Catriona A McNeil, Camilla Pramfalk, Sion A Parry, Wee Suan Low, Thomas Cornfield, Barbara A Fielding, Leanne Hodson

**Affiliations:** 1 Oxford Centre for Diabetes, Endocrinology and Metabolism, University of Oxford, Churchill Hospital, Oxford, United Kingdom; 2 Department of Public Health and Caring Sciences, Clinical Nutrition and Metabolism, Uppsala University, Uppsala, Sweden; 3 Division of Clinical Chemistry, Department of Laboratory Medicine, Karolinska Institute at Karolinska University Hospital Huddinge, Stockholm, Sweden; 4 Faculty of Health and Medical Sciences, University of Surrey, Guildford, United Kingdom; 5 Oxford NIHR Biomedical Research Centre, Churchill Hospital, Oxford, United Kingdom

**Keywords:** de novo lipogenesis, fatty acids, metabolism, human, triglycerides, lipogenic index, SCD, palmitoleic acid

## Abstract

**Background:**

Observational studies often infer hepatic de novo lipogenesis (DNL) by measuring circulating fatty acid (FA) markers; however, it remains to be elucidated whether these markers accurately reflect hepatic DNL.

**Objectives:**

We investigated associations between fasting hepatic DNL and proposed FA markers of DNL in subjects consuming their habitual diet.

**Methods:**

Fasting hepatic DNL was assessed using ^2^H_2_O (deuterated water) in 149 nondiabetic men and women and measuring the synthesis of very low-density lipoprotein triglyceride (VLDL-TG) palmitate. FA markers of blood lipid fractions were determined by gas chromatography.

**Results:**

Neither the lipogenic index (16:0/18:2n–6) nor the SCD index (16:1n–7/16:0) in VLDL-TG was associated with isotopically assessed DNL (*r* = 0.13, *P* = 0.1 and *r* = −0.08, *P* = 0.35, respectively). The relative abundances (mol%) of 14:0, 16:0, and 18:0 in VLDL-TG were weakly (*r* ≤ 0.35) associated with DNL, whereas the abundances of 16:1n–7, 18:1n–7, and 18:1n–9 were not associated. When the cohort was split by median DNL, only the abundances of 14:0 and 18:0 in VLDL-TG could discriminate between subjects having high (11.5%) and low (3.8%) fasting hepatic DNL. Based on a subgroup, FA markers in total plasma TG, plasma cholesteryl esters, plasma phospholipids, and red blood cell phospholipids were generally not associated with DNL.

**Conclusions:**

The usefulness of circulating FAs as markers of hepatic DNL in healthy individuals consuming their habitual diet is limited due to their inability to discriminate clearly between individuals with low and high fasting hepatic DNL.

## Introduction

In humans, the process whereby excess nonlipid precursors (e.g., sugars and protein) are synthesized to fat, de novo lipogenesis (DNL), primarily occurs in the cellular cytoplasm of the liver. It requires acetyl-CoA as a precursor and the principal building blocks to produce palmitoyl-CoA, the major quantitative product (1, 2). It is often suggested that enhanced DNL is related to metabolic diseases including nonalcoholic fatty liver disease (NAFLD), cardiovascular disease, and type 2 diabetes (TD2) (3–6), although it is unclear if this is a cause or an effect.

Hepatic DNL can be directly assessed in vivo in humans using stable-isotope tracers, where the appearance of the stable-isotope label [from deuterated water (^2^H_2_O) or [^13^C]acetate] is measured in very low-density lipoprotein (VLDL) triglyceride (TG) palmitate. Fasting hepatic DNL has been reported to be up to ∼10% in healthy adults (7–11) and significantly higher (up to 22%) in individuals with insulin resistance/NAFLD (7–9, 12).

There is a marked increase in the contribution of DNL to VLDL-TG after consumption of a high-carbohydrate diet when assessed by the linoleate-dilution method and ^13^C-acetate infusion (13). Intervention studies comparing low- with high-carbohydrate diets attribute observed changes in fatty acid (FA) composition of blood lipid fractions to DNL without having measured DNL (14). Although observational studies commonly use FA composition and/or FA ratios in various circulating lipid fractions to infer hepatic DNL (3–6), it has yet to be demonstrated that these FA markers are reflective of hepatic DNL during ambient/habitual dietary conditions. Therefore, we assessed if fasting hepatic DNL during habitual dietary conditions may be inferred from circulating FA markers in different lipid fractions.

## Methods

Subjects from 5 different cohorts were pooled, giving a total of 149 men and women who all had fasting DNL in VLDL-TG assessed with stable-isotopes; **Supplemental Figure 1**. A subgroup of 21 males also had newly synthesized palmitate assessed in total plasma TG, plasma cholesteryl esters (CE), plasma phospholipids (PL), and red blood cell (RBC) PL. The 21 healthy males were recruited from the Oxford BioBank (www.oxfordbiobank.org.uk) (15). All volunteers were considered nondiabetic and free from any known disease, were not taking medication known to affect lipid or glucose metabolism, and did not consume alcohol above recommended limits. Participants were considered to have low or high fasting hepatic DNL based on whether they were below or above the group median of 6.5%. All studies were approved by the respective Research Ethics Committees, and all subjects gave written informed consent. Some, but not all, of the data reported in this work constitute a reanalysis of previously published studies (16, 17).

### Body composition

Whole-body composition and fat distribution were measured using dual-energy X-ray absorptiometry (18).

### Measurements of DNL

Before the measurement of fasting hepatic DNL, subjects were asked to avoid alcohol and strenuous exercise, and a fasting blood sample was taken to measure background isotopic enrichment in plasma water and VLDL-TG. The evening before the study day (∼12 h before assessment), subjects consumed ^2^H_2_O (3 g/kg body water) and continued to consume enriched water (2.5 g per 500 mL of water) over the course of the evening, in order to achieve and maintain a plasma water enrichment of 0.3%. Based on the previous work by Diraison et al. (19), which clearly demonstrated that within 4 h of a participant being given loading doses of ^2^H_2_O, an enrichment plateau of deuterium in plasma water was achieved, it is likely that subjects in the present study achieved and maintained an enrichment plateau of deuterium in plasma water. Subjects were advised to consume a low-fat dinner the evening before measurement of DNL. After an overnight fast, subjects came to the Clinical Research Unit, and a blood sample was taken.

### Analytical methods

Whole blood was collected into heparinized syringes (Sarstedt), and plasma was rapidly separated by centrifugation at 4°C for measurement of plasma FA composition, plasma metabolites were analyzed enzymatically, and insulin was measured by radioimmunoassay [Millipore (UK) Ltd] (20). After removal of plasma, the upper buffy coat was removed, and the packed erythrocytes were washed as described (21) and stored at −80°C for the analysis of RBC total PL FAs.

Separation of the VLDL-rich fraction (S_f_20–400) was made by sequential flotation using density gradient ultracentrifugation (22, 23), although for 1 study (16), VLDL_1_ and VLDL_2_ were isolated from plasma using density-gradient ultracentrifugation (24). Because the calculated DNL in VLDL_1_ and VLDL_2_ were similar, we have used the data for DNL in VLDL_1_.

### Fatty acid composition, indexes, and isotopic enrichment

FA methyl esters (FAMEs) were prepared from the VLDL-TG fraction (22, 23) and plasma PL, CE, TG, and RBC (25–27). FA relative abundance (mol%) was determined by gas chromatography (GC) (28).

The ratio of palmitic acid (16:0) to linoleic acid (18:2n–6) [lipogenic index (29)] in the respective blood lipid fractions was used as an index of hepatic DNL and the ratio of palmitoleic acid (16:1n–7) to palmitic acid (16:0) (SCD index) as a marker of stearoyl-CoA desaturase (SCD) activity (30).

Fasting hepatic DNL was assessed based on the incorporation of deuterium from ^2^H_2_O in plasma water (Finnigan GasBench-II, ThermoFisher Scientific) into VLDL-TG palmitate along with the incorporation of deuterium from ^2^H_2_O in plasma water into total plasma TG, CE, PL, and RBC PL palmitate using GC-MS with monitoring ions with mass-to-charge ratios (*m/z*) of 270 (M + 0) and 271 (M + 1) (31). The percentage “DNL” represents the synthesis of palmitate from nonlipid precursors (1).

### Statistics

Data were analyzed using JMP 13.1.0 (SAS Institute Inc.). Data are presented as median (IQR) or mean ± SD. Comparisons of DNL with the different lipid fraction parameters were assessed using a Wilcoxon-signed rank test. Differences between subgroups were analyzed using the *t*-test or Mann–Whitney *U*-test. Associations between variables were carried out using Spearman's rank correlation coefficient. Multiple regression analyses were performed with BMI, visceral fat, and HOMA-IR as covariates. *P* < 0.05 was considered statistically significant. The primary outcome of the present study was to investigate the associations between fasting hepatic DNL and proposed fatty acid markers of DNL in VLDL-TG.

## Results

**FIGURE 1:**
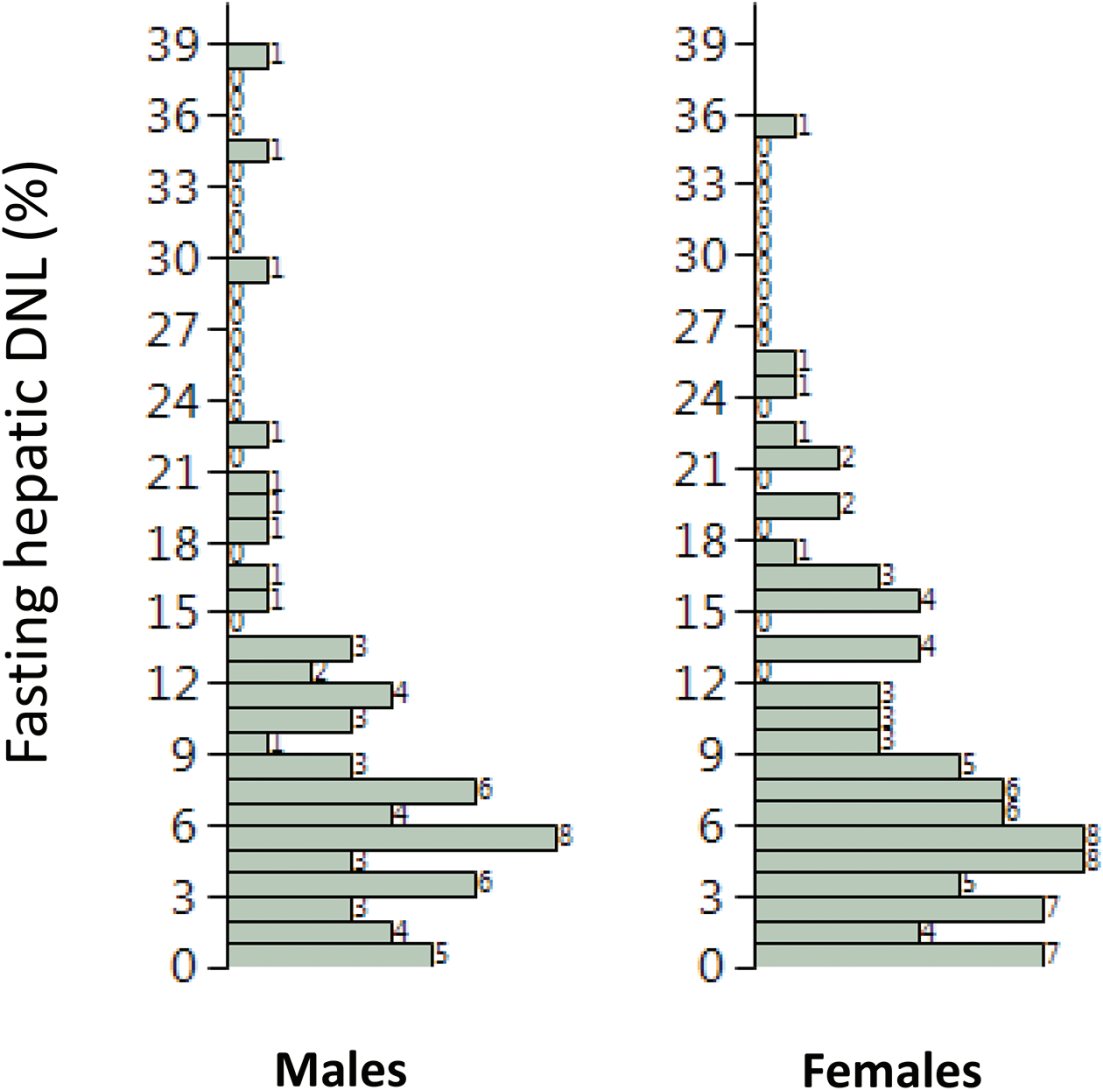
Distribution of liver fat in males and females. DNL, de novo lipogenesis.

Subject characteristics are shown in **Table 1** and the distribution of DNL in **Figure 1**. Fasting hepatic DNL was not different (*P* = 0.83) between males [6.71% (3.87–11.85) median (interquartile range)] and females [6.23% (3.34–11.40) median (interquartile range)]. The median age and BMI of the whole cohort were 47 y (range 23–67 y) and 27.3 kg/m^2^ (range 19.5–39.5 kg/m^2^), and the fasting hepatic DNL was 6.5% (range 0.1–38.7%). We classified individuals as having high [*n* = 74 (*n* = 41 females)] and low [*n* = 75 (*n* = 44 females)] fasting hepatic DNL based on the median of 6.5%. There was no difference in age between the groups, but the high DNL group had significantly (*P* < 0.05 for all) higher BMI, plasma insulin, and TG concentrations than the low DNL group (Table 1). The biochemical and clinical variables most strongly associated with fasting hepatic DNL were fasting insulin (*r* = 0.39, *P* = 0.0001), fasting TG (*r* = 0.41, *P* = 0.0001), and waist circumference (*r* = 0.35, *P* = 0.0001). BMI (*r* = 0.21, *P* = 0.009), body fat (*r* = 0.20, *P* = 0.02), and visceral fat (*r* = 0.21, *P* = 0.027) were only weakly associated. Age, glucose, nonesterified fatty acids, and total cholesterol were not associated, whereas HDL cholesterol was inversely associated (*r* = −0.26, *P* = 0.001).

**TABLE 1:** Characteristics of study participants^1^

	All subjects (*n* = 149)	Low DNL (*n* = 75)	High DNL (*n* = 74)
Male/female	64/85	31/44	33/41
Age, y	47.3 ± 8.4	47.3 ± 7.9	47.7 ± 8.9
BMI, kg/m^2^	27.3 ± 3.9	26.4 ± 3.6	28.1 ± 4.1**
Waist, cm	92 ± 13	88 ± 12	95 ± 13***
Body fat, %	34 ± 7	32 ± 7	35 ± 7**
Visceral fat, kg	1.1 (0.5–2.0)	0.8 (0.4–1.9)	1.4 (0.6–2.3)*
Homeostatic model assessment of insulin resistance	2.5 (1.9–3.5)	2.4 (1.7–2.9)	3.0 (2.1–4.6)***
Glucose, mmol/L	5.2 ± 0.5	5.2 ± 0.5	5.3 ± 0.5
Insulin, mU/L	11.1 (9.2–13.6)	10.3 (7.7–12.2)	12.7 (9.8–20.1)***
Total cholesterol, mmol/L	5.2 ± 1.0	5.2 ± 1.0	5.3 ± 0.9
HDL cholesterol, mmol/L	1.4 ± 0.4	1.4 ± 0.4	1.3 ± 0.4*
Nonesterified fatty acids, µmol/L	512 ± 183	522 ± 187	503 ± 181
Triglyceride, mmol/L	1.0 (0.7–1.9)	0.9 (0.6–1.3)	1.5 (0.9–2.1)***
DNL, %	6.5 (3.8–11.5)	3.8 (1.8–5.2)	11.5 (8.5–16.5)***

^1^Data are presented as means ± SDs or medians (IQR). *P < 0.05; **P < .001; ***P < 0.001 low DNL compared with high DNL. Differences were analyzed using the *t*-test or Mann–Whitney *U*-test. Low/High DNL groups based on lower/higher than median DNL in all subjects. BMI, body mass index; DNL, de novo lipogenesis.

### Fasting hepatic DNL and associations with fatty acid markers

**FIGURE 2:**
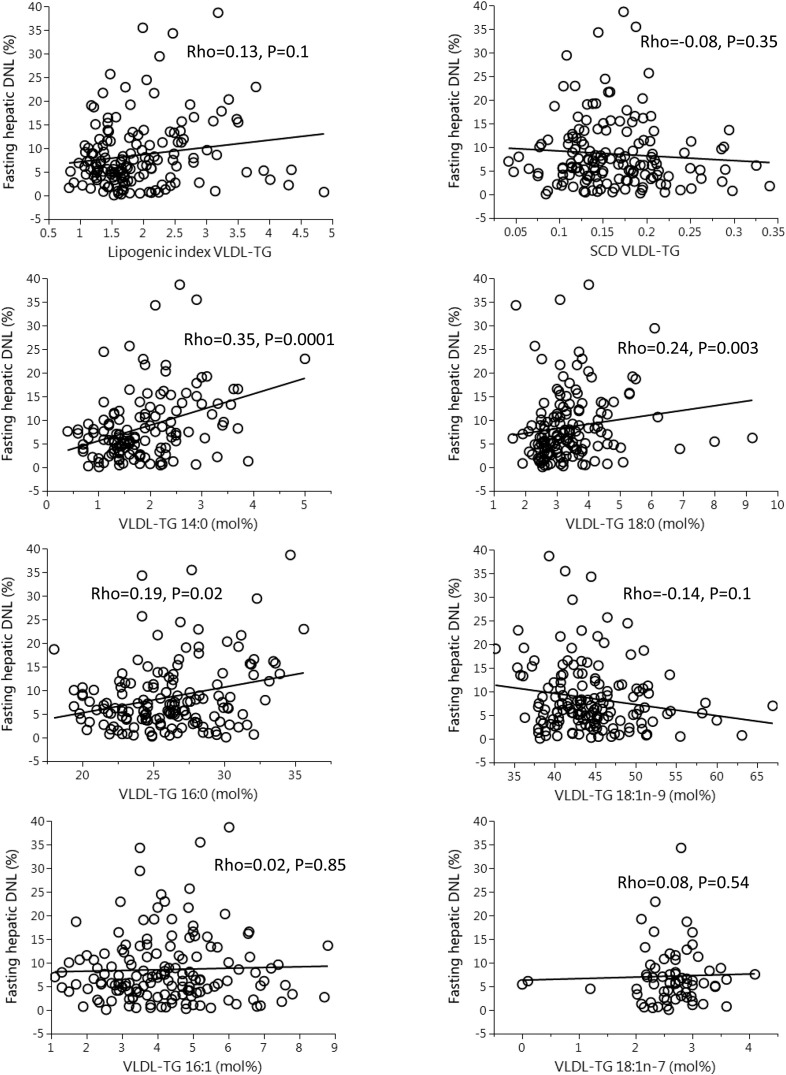
Correlations (Spearman rho) between fatty acid markers in VLDL-TG and fasting hepatic DNL. *n* = 149 for all except 14:0 (*n* = 134) and 18:1n–7 (*n* = 67). DNL, de novo lipogenesis; Rho, rank correlation coefficient; SCD, stearoyl-CoA desaturase; VLDL-TG, very low-density lipoprotein-triglyceride.

Because FA ratios (lipogenic and SCD index) and/or specific FAs are often used to infer hepatic DNL, we assessed whether these were associated with DNL in VLDL-TG. We found that neither the lipogenic index nor the SCD index was significantly associated with DNL in VLDL-TG (**Figure 2**). Because the FAs 14:0, 16:1n–7, 18:0, 18:1n–9, and 18:1n–7 have been suggested to be part of the DNL pathway (3, 4, 6, 9), we assessed the association between DNL and the abundance of specific FAs in VLDL-TG (Figure 2). We found the relative abundance (mol%) of 14:0, 16:0, and 18:0 to be weakly/moderately positively associated with DNL, whereas 16:1n–7, 18:1n–9, and 18:1n–7 were not associated with DNL (Figure 2).

### Fasting DNL and fatty acid markers: subgroup analysis

**FIGURE 3:**
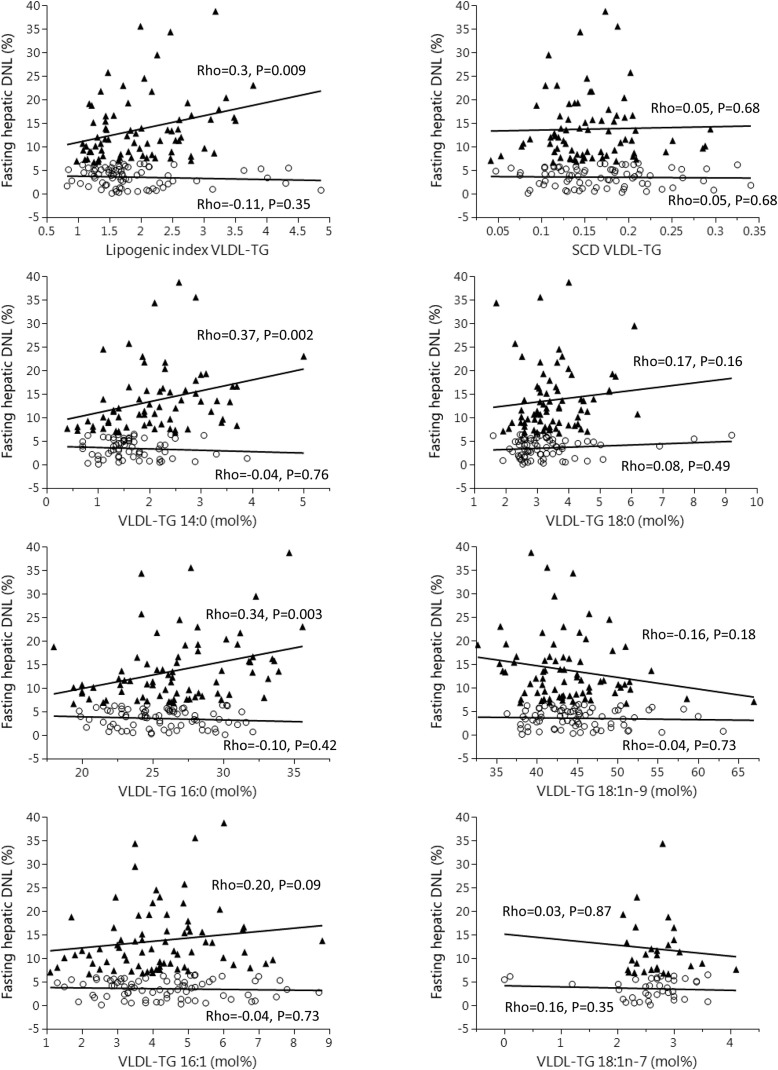
Correlations (Spearman rho) between fatty acid markers in VLDL-TG and fasting hepatic DNL in subgroups split by median fasting hepatic DNL. “High DNL” denoted by filled triangle, “Low DNL” denoted by open circle. *n* = 74/75 for High/Low DNL except for 14:0 (*n* = 67/67) and 18:1n–7 (*n* = 29/38). DNL, de novo lipogenesis; Rho, rank correlation coefficient; SCD, stearoyl-CoA desaturase; VLDL-TG, very low-density lipoprotein-triglyceride.

To investigate if FA markers could be used to discriminate between subjects with high DNL and those with low DNL, we divided the cohort by median DNL and compared the groups. The VLDL-TG lipogenic and SCD indexes were not different between individuals classified as having high and those with low fasting hepatic DNL (**Table 2**). Proportions of 14:0 and 18:0 in VLDL-TG were higher in subjects with higher than in those with lower DNL, whereas proportions of 16:0, 16:1n–7, 18:1n–9, and 18:1n–7 were not different between groups (Table 2). We assessed the associations between DNL and FA markers in the respective groups and found that correlations were stronger in subjects classified as having higher than in those with lower hepatic DNL (**Figure 3**).

**TABLE 2:** Fasting fatty acid composition (mol %) of very low-density lipoprotein-triglyceride in individuals classified as having low and high fasting hepatic DNL^1^

	Low DNL (*n* = 75)	High DNL (*n* = 74)
Lipogenic Index (16:0/18:2n–6)	1.66 ± 0.09	1.87 ± 0.08
Stearoyl-CoA desaturase Index (16:1n–7/16:0)	0.15 ± 0.01	0.16 ± 0.01
14:0	1.5 ± 0.07	2.1 ± 0.11***
16:0	25.5 ± 0.36	26.4 ± 0.48†
16:1n–7	4.2 ± 0.19	4.1 ± 0.18
18:0	3.0 ± 0.14	3.4 ± 0.11**
18:1n–9	44.5 ± 0.62	43.3 ± 0.64
18:1n–7	2.7 ± 0.12	2.7 ± 0.08
18:2n–6	15.3 ± 0.47	14.3 ± 0.43

^1^Data presented as medians ± SEMs. †*P* = 0.08, **P < 0.001; ***P < 0.001 low DNL compared with high DNL. Differences were analyzed using Mann–Whitney U-test. Low/high DNL groups based on lower/higher than median DNL in all subjects. DNL, de novo lipogenesis.

Because the subgroups differed in characteristics (Table 1), the results could potentially be confounded; therefore we also performed a regression analysis in the full cohort adjusting for BMI, visceral fat, and insulin resistance (HOMA-IR) (**Table 3**). The proportion of 14:0 in VLDL-TG remained significantly associated with hepatic DNL, such that a 1% increase in VLDL-TG 14:0 abundance was associated with a 2.59% increase in DNL. The proportion of 18:0 in VLDL-TG was no longer significant after adjustment, and the other FA markers remained nonsignificant (Table 3). Although the abundances of 14:0, 16:0, and 18:0 in VLDL-TG were significantly associated with DNL in unadjusted analyses (Figure 2), the diagnostic values were poor. If “high DNL” (top quartile) was “diagnosed” using FA abundance (top quartile), the predictive values were 52% for 14:0, 41% for 16:0, and 38% for 18:0, respectively. Similarly, if “low DNL” (bottom quartile) was “diagnosed” using FA abundance (bottom quartile), the predictive values were 32% for 14:0, 24% for 16:0, and 37% for 18:0, respectively.

**TABLE 3:** Multiple regression analysis between hepatic de novo lipogenesis and fatty acid markers after adjusting for potential confounding variables (BMI, visceral fat, and homeostatic model assessment of insulin resistance)

	*β*	*P* value
Lipogenic Index (16:0/18:2n–6)	1.69	0.11
Stearoyl-CoA desaturase Index (16:1n–7/16:0)	−2.22	0.87
14:0	2.59	0.008
16:0	0.34	0.094
16:1n–7	0.25	0.59
18:0	0.32	0.61
18:1n–9	−0.1	0.44
18:1n–7	0.33	0.78
18:2n–6	−0.35	0.09

### Use of other blood lipid fractions to assess fasting DNL

**FIGURE 4:**
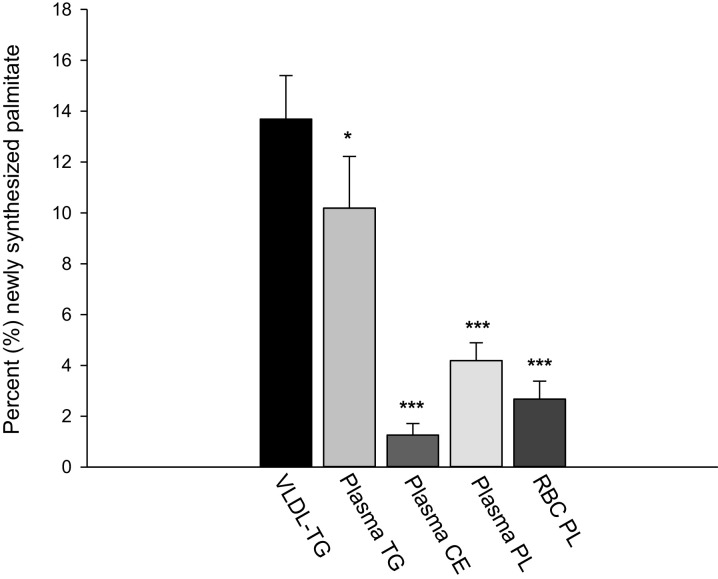
Percentage newly synthesized palmitate in circulating VLDL-TG, total plasma TG, plasma CE, plasma PL, and RBC PL. *n* = 21 men for all fractions. *P < 0.05, ***P < 0.001 compared with VLDL-TG; analyzed using Wilcoxon-signed rank test. CE, cholesteryl esters; DNL, de novo lipogenesis; PL, phospholipids; RBC, red blood cells; TG, triglyceride; VLDL-TG, very low-density lipoprotein-triglyceride.

As plasma VLDL-TG is not measured in a large proportion of studies, we investigated whether other more commonly used blood lipid fractions could be used to assess fasting hepatic DNL. In a subgroup of *n* = 21 males, DNL was measured in VLDL-TG, total plasma TG, CE, PL, and RBC PL while consuming their habitual diet. When compared with DNL in VLDL-TG, which was 13.7 ± 7.9% (mean ± SD), we found it to be significantly lower in all other fractions: 10.2 ± 9.3% in total plasma TG, 1.3 ± 2.1% in plasma CE, 4.2 ± 3.2% in plasma PL, and 2.7 ± 3.2% in RBC PL, all *P* ≤ 0.002 compared with VLDL-TG (**Figure 4**). Finally, we investigated the associations between the proposed FA markers in these other fractions with DNL and found no significant associations for the lipogenic or SCD indexes in any fraction (**Table 4**). Specific FAs were generally not associated with DNL, except for 18:0 showing positive associations (Figure 4).

**TABLE 4:** Correlation coefficients between hepatic de novo lipogenesis and fatty acid markers in other blood lipid fractions^1^

Fasting hepatic de novo lipogenesis	Plasma triglycerides	Plasma cholesteryl esters	Plasma phospholipids	Red blood cell phospholipids
Lipogenic Index (16:0/18:2n–6)	0.16	0.10	−0.04	0.24
Stearoyl-CoA desaturase Index (16:1n–7/16:0)	−0.15	0.22	0.04	−0.23
14:0	0.21	−0.27	0.13	−0.01
16:0	0.12	0.04	−0.35	0.09
16:1n–7	−0.07	0.12	−0.03	−0.25
18:0	0.46	0.46*	0.73*	0.64*
18:1n–9	−0.17	0.24	0.57*	0.27
18:1n–7	−0.50*	−0.23	−0.34	0.02

^1^ *n* = 21 men for all fractions. Correlation coefficients are Spearman rho. *P < 0.05. Rho, rank correlation coefficient.

## Discussion

Ideally, hepatic DNL is measured using stable-isotope methodologies, but, for practical reasons, as proxy markers circulating FAs or FA ratios are often measured. However, it remains unclear how reflective these markers are of hepatic DNL during habitual dietary conditions, so we investigated the association between fasting hepatic DNL (assessed in VLDL-TG using stable-isotope methodologies) and circulating FA markers that are often used to infer hepatic DNL in healthy individuals consuming their habitual diet. We did not find any strong associations between FA markers and DNL, and diagnostic values were poor, suggesting that fasting hepatic DNL in subjects consuming their habitual diet is not reliably inferred from commonly used FA proxies.

DNL is an insulin-mediated process, and we have previously reported higher DNL in hyperinsulinemic than in normoinsulinemic individuals (11). In the present study, plasma insulin concentrations were positively associated with fasting DNL.

Large observational studies have assessed the relation between individual FAs and FA ratios with outcomes such as NAFLD or T2D (3, 6) and found that the RBC lipogenic index and 16:1n–7 tended to be positively associated with a fatty liver index (FLI) (3). In the present study, we found no association between fasting DNL in VLDL-TG and the lipogenic and SCD indexes or abundance of 16:1n–7 in VLDL-TG. The lack of association between DNL and the lipogenic index is in agreement with Lee et al. (9), although, in contrast to our findings, they observed strong positive associations between DNL, the SCD index, and the relative abundance of 16:1n–7 in VLDL-TG (9). Dissociation between DNL and the SCD index has previously been reported (11, 16, 32). We have previously calculated an isotopic desaturation index in VLDL-TG and found a significant correlation with DNL (16), suggesting the isotopic index may be more relevant as a marker of overnight fasting FA desaturation than a nonisotopic index.

Our results are partly in agreement with earlier work (13, 29). In a study of 10 healthy subjects, Hudgins et al. (29) fed liquid formula diets either high (40% total energy (TE), *n* = 3) or low (10% TE, *n* = 7) in fat, with matched FA composition, for 25 d. They found that the proportions of 14:0, 16:0, and 16:1 in VLDL-TG were all higher on the low-fat diet than on the high-fat diet, whereas the proportions of 18:0, 18:1n–9, and 18:1n–7 did not appear to be differentially affected. Hudgins et al. (13) later replicated some of this work using solid-food diets (given for 2 wk) and found that the proportion of 16:0, but not 18:1n–9, was higher in VLDL-TG after a low-fat (10% TE) than after a high-fat (30% TE) diet in both lean and obese subjects. These data suggest that in order to see a change in FA composition that would be reflective of hepatic DNL, there needs to be a provocation or stimulus, such as a low-fat, high-carbohydrate diet (34).

In humans, tissue and blood FAs are reflective of dietary FA intake (35), but the proportion of carbohydrate in the diet can influence hepatic DNL (2, 13, 29). We previously found that short-term consumption (3 d) of a high-carbohydrate diet increased both the lipogenic and SCD indexes in VLDL-TG compared with a lower carbohydrate diet (33). Although changes in dietary fat composition are reflected by the FA composition of blood lipid fractions within days (25), it remains unclear how rapidly changes in DNL FAs are reflected. Compared with VLDL-TG, we found significantly lower DNL in plasma TG, CE, PL, and RBC PL, which may be due to longer turnover times. VLDL-TG contributes greater than 50% of total plasma TG in healthy individuals (36); however, DNL in this fraction was significantly lower than VLDL-TG, which may be explained by dilution from TG present in other circulating lipoproteins. We found evidence for DNL in the plasma PL fraction, and although de novo FAs have been suggested to be incorporated into PL to a greater extent than exogenous FAs (37), the complexities of PL synthesis along with known enzyme specificities make it unclear if measuring markers of de novo FAs would clearly discriminate between individuals with high and low hepatic DNL.

DNL was lowest in plasma CE, which may be due to the specificities of the intracellular enzyme responsible for CE formation (35). The CE fraction comprises <5% of total liver FAs (38), and because CE formation is not liver-specific, it is likely that the plasma CE fraction is not reflective of hepatic DNL. It has previously been suggested under lipogenic conditions that only 2–3% of newly synthesized palmitate is secreted as VLDL-CE and -PL (39). In contrast, RBCs are incapable of de novo PL synthesis, chain elongation, or desaturation (40, 41), so the appearance of any newly synthesized palmitate would occur by direct exchange of phosphatidylcholine (PC) from plasma lipoproteins to the RBC membrane (42) and acylation of lysophospholipids, which are formed in the membrane or derived from surrounding plasma (43). Changes in dietary FA intakes are reflected in the FA composition of RBC PC within days (44), so it is plausible that the appearance of newly synthesized palmitate occurred as a result of direct and rapid FA exchange.

Our study has some limitations. Dietary intakes were not standardized or assessed on the days before the study visit, and it is possible that some subjects made acute dietary changes before the study day; however, this setting is the reality for the majority of cohorts inferring DNL from circulating FA markers. Because we measured DNL after an overnight fast, we were unable to assess the synthesis of other DNL FAs as carried out by Wilke et al. (2), which would be of interest to do because it would help to clarify what specific FAs may be useful surrogate markers of hepatic DNL in individuals consuming their habitual diet. The labeling period may have been too short to accurately quantify newly synthesized palmitate in lipid fractions other than VLDL-TG or total plasma TG due to slower turnover. A longer labeling time could be argued for the assessment of DNL in VLDL-TG due to a potential delay in secretion of newly formed palmitate. However, recirculation of labels might result in exaggerated values of DNL; our protocol for the assessment of DNL is in line with previous studies (8, 31, 45). It has recently been reported (46) that labeling with ^2^H_2_O for 7–14 d gives higher DNL values than the shorter-term labeling approaches more commonly used. The duration of labeling is a trade-off between cost, feasibility, and the ability to detect delayed secretion of newly formed palmitate, and the “optimal” duration for labeling should be further explored. Finally, we only assessed nondiabetic individuals, so it is plausible that our findings would be different if we had studied other cohorts such as individuals with T2D.

The use of FA markers as indirect measurements of DNL from a single fasting blood sample in participants consuming their habitual diet may not discriminate reliably between individuals with low and high hepatic DNL. Therefore, to disentangle the origin (dietary or endogenously synthesized) of FAs in blood lipid fractions, gold-standard stable-isotope tracer methods are required.

## Supplementary Material

nqy304_Supplemental_File

## Notes

This study was funded by the British Heart Foundation FS/11/18/28633 (LH), FS/15/56/31645 (LH), PG/09/003 (BAF), Biotechnology and Biological Sciences Research Council UK BB/N005600/1, and BB/N015665/1 (LH). This work was supported by the NIHR Biomedical Research Centre, Oxford. The Oxford BioBank (www.oxfordbiobank.org.uk) is also part of the NIHR National Bioresource, which supported the recalling process of the volunteers.

Supplemental Figure 1 is available from the “Supplementary data” link in the online posting of the article and from the same link in the online table of contents at https://academic.oup.com/ajcn/.

FR and CAM contributed equally.

Abbreviations used:

CE: cholesteryl ester
DNL: de novo lipogenesis
FA: fatty acid
FAME: fatty acid methyl ester
FLI: fatty liver index
GC: gas chromatography
HOMA-IR: homeostatic model assessment of insulin resistance
MS: mass spectrometry
NAFLD: nonalcoholic fatty liver disease
PC: phosphatidylcholine
PL: phospholipid
RBC: red blood cell
SCD: stearoyl-CoA desaturase
T2D: type 2 diabetes
TG: triglyceride
VLDL: very low-density lipoprotein
