# Supplementary material for: Fasting hepatic de novo lipogenesis is not reliably assessed using circulating fatty acid markers
Source: Am J Clin Nutr. 2019 Feb 5;109(2):260–8. doi: 10.1093/ajcn/nqy304 (PMC6367991; doi:10.1093/ajcn/nqy304)
Supplement: nqy304_Supplemental_File [file nqy304_supplemental_file.zip › Revised_Supplemental Figure 1 - Flowchart.pptx]

## Slide 1
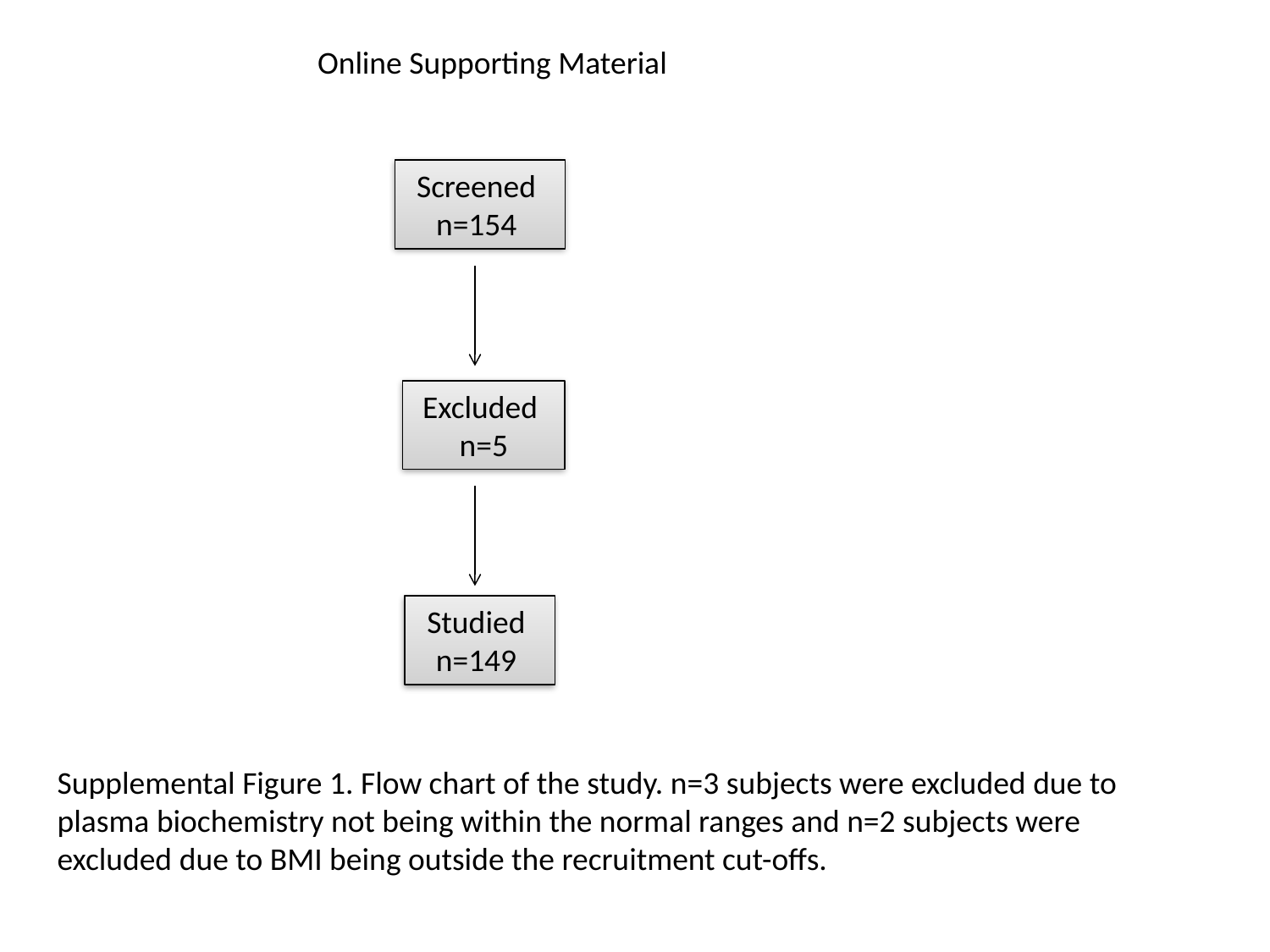

Online Supporting Material
Screened
n=154
Excluded
n=5
Studied
n=149
Supplemental Figure 1. Flow chart of the study. n=3 subjects were excluded due to plasma biochemistry not being within the normal ranges and n=2 subjects were excluded due to BMI being outside the recruitment cut-offs.
